# Site-Specific Integration of Foreign DNA into Minimal Bacterial and Human Target Sequences Mediated by a Conjugative Relaxase

**DOI:** 10.1371/journal.pone.0031047

**Published:** 2012-01-23

**Authors:** Leticia Agúndez, Coral González-Prieto, Cristina Machón, Matxalen Llosa

**Affiliations:** Departamento de Biología Molecular, Universidad de Cantabria (UC) and Instituto de Biomedicina y Biotecnología de Cantabria, UC-CSIC-SODERCAN, Santander, Spain; Naval Research Laboratory, United States of America

## Abstract

**Background:**

Bacterial conjugation is a mechanism for horizontal DNA transfer between bacteria which requires cell to cell contact, usually mediated by self-transmissible plasmids. A protein known as relaxase is responsible for the processing of DNA during bacterial conjugation. TrwC, the relaxase of conjugative plasmid R388, is also able to catalyze site-specific integration of the transferred DNA into a copy of its target, the origin of transfer (*oriT*), present in a recipient plasmid. This reaction confers TrwC a high biotechnological potential as a tool for genomic engineering.

**Methodology/Principal Findings:**

We have characterized this reaction by conjugal mobilization of a suicide plasmid to a recipient cell with an *oriT*-containing plasmid, selecting for the cointegrates. Proteins TrwA and IHF enhanced integration frequency. TrwC could also catalyze integration when it is expressed from the recipient cell. Both Y18 and Y26 catalytic tyrosil residues were essential to perform the reaction, while TrwC DNA helicase activity was dispensable. The target DNA could be reduced to 17 bp encompassing TrwC nicking and binding sites. Two human genomic sequences resembling the 17 bp segment were accepted as targets for TrwC-mediated site-specific integration. TrwC could also integrate the incoming DNA molecule into an *oriT* copy present in the recipient chromosome.

**Conclusions/Significance:**

The results support a model for TrwC-mediated site-specific integration. This reaction may allow R388 to integrate into the genome of non-permissive hosts upon conjugative transfer. Also, the ability to act on target sequences present in the human genome underscores the biotechnological potential of conjugative relaxase TrwC as a site-specific integrase for genomic modification of human cells.

## Introduction

Bacterial conjugation is a mechanism for horizontal gene transfer among bacteria. By this process, a DNA molecule of any origin and length can be transferred to a recipient cell, if it contains an origin of transfer (*oriT*); the conjugative machinery can be provided in *trans*. Under laboratory conditions, conjugation to eukaryotic cells has been reported, from bacteria to yeast [1], plants [2] and mammalian cells [3].

Mechanistically, bacterial conjugation can be viewed as a plasmid DNA replication system linked to a secretion channel [4], leading to horizontal rather than vertical transmission of the plasmid. Accordingly, there are three functional modules in the conjugative machinery [5]:

- the relaxosome, a nucleoprotein complex required for plasmid DNA processing, which is related to rolling-circle replication systems. This complex includes a relaxase protein, often together with accessory proteins which assist its function, and the *oriT*, which is the only DNA sequence required *in cis* for DNA transfer.
- the Type IV secretion system (T4SS), a multiprotein complex which forms the transmembranal channel for substrate secretion.
- the coupling protein (T4CP), responsible for linking the other two modules by protein-protein interactions.

The relaxase nicks the *oriT* in the DNA strand which is going to be transferred and remains covalently bound to the 5′ end; current models for conjugative DNA transfer propose that this nucleoprotein complex is secreted to the recipient cell through the T4SS, followed by active DNA pumping by the ATPase activity of the T4CP [4]. Once in the recipient cell, the relaxase is functional [6], and presumably recircularizes the transferred DNA.

The relaxosome of the conjugative plasmid R388 comprises the *oriT* (*oriTw*), the relaxase-helicase TrwC, and two helper proteins, R388-encoded TrwA and host-encoded IHF (integration host factor) [7]. The minimal sequence of the *oriTw* essential for *in vivo* mobilization is only 17 bp in length; *in vitro*, it was shown that the sequence 6 nt 5′ and 2 nt 3′ to the *nic* site (6+2) is absolutely required for TrwC binding and for DNA nicking and strand transfer reactions on oligonucleotides [8]. The helper protein TrwA binds to *oriTw*; it acts as a transcriptional repressor of the *trwABC* operon and also increases TrwC nicking activity on *oriTw* [9], [10]. IHF is a host protein which binds specifically to *oriTw*. IHF has been proposed to have a negative effect on TrwC nicking activity, but not TrwC binding [7].

TrwC relaxase activity, responsible for DNA strand cleaving and transfer activities on supercoiled or single-stranded substrates, is contained in its N-terminal 293 residues (N293) [11]. This domain contains catalytic residues Y18 and Y26, which act sequentially [12]: Y18 is the only tyrosine able to act on supercoiled plasmid substrates and so it is responsible for the initial nicking reaction, while Y26 would catalyze the final strand-transfer reaction. The DNA helicase, ATPase and dimerization activities of TrwC are located in the C-terminal residues (C774) [13].

Apart from its role in conjugation, TrwC has been shown to act as a site-specific recombinase between two *oriTw* copies repeated in tandem [14]. This reaction occurs in the absence of conjugation, and thus in the absence of single stranded intermediates. This ability of TrwC is not a general feature of relaxases; TraI of plasmid F, which shares a similar 3D catalytic fold, is not able to catalyze this reaction [15]. TrwC-mediated site-specific recombination is strongly enhanced by TrwA [15]. Host factors which affect DNA topology, such as IHF or transcription through the *oriTw*, affect the reaction [16]. One *oriTw* copy could be narrowed to the core sequence of 17 bp (14 nt 5′+3 nt 3′of *nic*) and still support TrwC-mediated recombination efficiently [15]. A search for possible natural targets sequences for TrwC in the human genome demonstrated that there are at least two possible natural targets on which TrwC can act as a recombinase [17]. The recombinase domain of TrwC locates to the nucleus in human cells, and, by random mutagenesis, a TrwC mutant which enters the nucleus was also obtained [17]. Interestingly, TrwC can catalyze site-specific integration of the incoming DNA into an *oriTw*-containing plasmid in the recipient cell [6]. Overall, these data underscore the biotechnological potential of TrwC as a site-specific integrase which could be introduce into human cells for genomic modification.

In this work we have modified the integration assay in order to address the DNA and protein requirements of the reaction. Our results suggest a model for TrwC-mediated site-specific integration. We have obtained TrwC-mediated site-specific integration on minimal *oriTw* and human target sequences and into the *Escherichia coli* chromosome, underlying the biological significance of this reaction and the potential of TrwC for biotechnological purposes.

## Results

### Optimization of the integration assay

**Figure 1:**
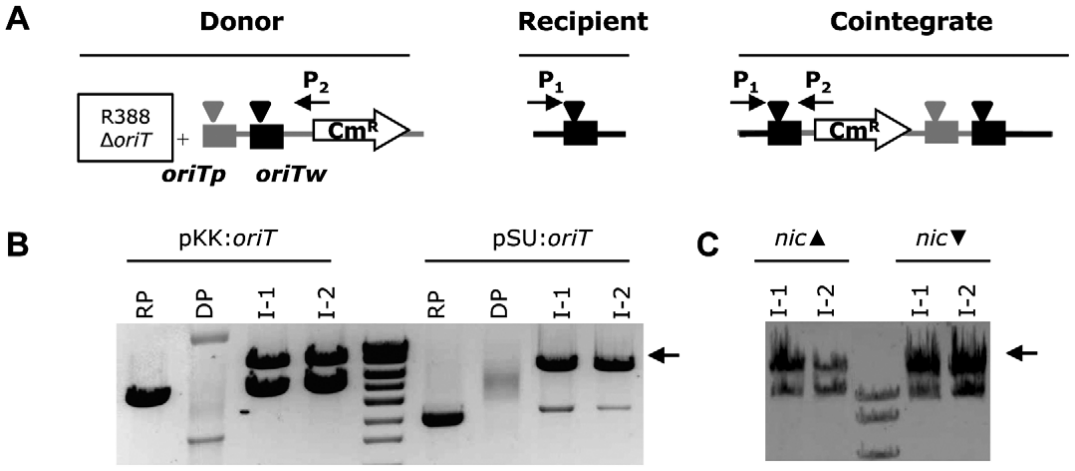
Site-specific integration assays with different recipient plasmids. **A**. Scheme of the assay and the cointegrate molecule obtained. The suicide plasmid is represented with a grey line and the recipient plasmid with a black line. The *nic* site is indicated by an arrowhead. P1 and P2, oligonucleotides used in the PCR reaction to detect the cointegrates. **B and C**. Restriction analysis with enzymes that cut only once in the recipient plasmid. *NdeI* was used for integrants obtained in pKK:*oriT* (in **B**, to the left of the MW marker), *XcmI* for integrants in pSU:*oriT* (in **B**, to the right of the MW marker), and *BstEII* for integrants in pLA58 and pLA59 (in **C**, *nic*▴ and *nic*▾, respectively). The cointegrate is indicated with an arrow. DP, donor plasmid; RP, recipient plasmid. I1-I2, DNA from two independent integrants obtained on each RP. Sizes in kb of the MW marker from top of the gel: 10-8.0-6.0-5.0-4.0-3.0-2.5.

In earlier work, the ability of TrwC to catalyze site-specific integration was assayed by mobilization of a suicide plasmid to an *oriTw*-containing recipient strain, in a *recA* background [6]. The assay is depicted in **Figure 1A**. The suicide plasmid pR6K::*oriTp oriTw*, containing the *oriTs* of plasmids R388 (IncW) and RP4 (IncP), is mobilized by the conjugative system of plasmid R388 from donor strain CC118 λpir (coding for the Pir protein which allows replication of the R6K replicon) to a recipient strain which contains a plasmid harbouring another *oriTw* copy, where integration takes place. The suicide plasmid cannot replicate in the recipient strain. The integrants are selected in Cm plates, the resistance conferred by the suicide plasmid, and integration subsequently confirmed by PCR amplification of a specific region of the cointegrate molecule with primers P1 and P2 (Fig. 1A). The published integration frequency reached with this assay was 5.4×10^−6^ integrants per donor. It was also reported that the reaction was TrwC-dependent, since no integration events were detected when, in a similar assay, the suicide plasmid was mobilized by the RP4 conjugative relaxase [6].

To improve the integration reaction, we used a different Pir donor strain, II1, which can be handled at 37°C since it is devoid of the λ prophage. As recipient plasmids, we assayed two different replicons: pKK::*oriTw* (pMB8 replicon) and pSU::*oriTw* (p15A replicon). With either recipient plasmid, a significant improvement in the integration frequency was obtained (**Table 1**): ca. 2 logs with pKK::*oriTw* and 1 log with pSU::*oriTw*, compared to earlier work. However, we observed a residual level of integration (2.6×10^−7^ integrants/donor) in the absence of TrwC when using pKK::*oriTw*, for unknown reasons, while no integrants were found in the absence of TrwC when the pSU:*oriTw* recipient plasmid was used (Table 1). Thus, the pSU::*oriTw* recipient plasmid rendered the best integration frequency maintaining TrwC specificity and was used as the recipient plasmid for subsequent integration assays.

**Table 1:** TrwC-mediated site-specific integration into different recipient plasmids.

	Recipient				
Donor	DH5αλpir^1^	pKK	pKK::*oriTw*	pSU::*oriTw*	pSU::*oriTp*
**II1 (pCIG1077+pR6K::** ***oriTporiTw*** **)**	1.77	<7×10^−6^	2.4×10^−4^	5.3×10^−5^	<2.8×10^−8^
**S17.1 λpir (pR6K::** ***oriTp oriTw)***	0.32	<4.9×10^−7^	2.6×10^−7^	<2.2×10^−8^	<2.2×10^−8^

Integration frequencies expressed as integrants/donor. Integrants were confirmed by PCR amplification of a region of the cointegrate, as explained in Experimental Procedures. The recipient strain was DH5α.

1 DH5α λ*pir* was used as an internal control for conjugation efficiency, expressed as transconjugants/donor.

We tested the ability of RP4_TraI to promote site-specific integration under similar assay conditions, mobilizing the suicide plasmid by this relaxase to a recipient which contained pSU::*oriTp*. No integrant colonies were found (Table 1, last line, last column), suggesting that RP4_TraI relaxase is not able to catalyze site-specific integration of an incoming DNA. However, transfer of TraI-RP4 to the recipient cell has not been proved experimentally so far. Thus, we cannot discard that the absence of integration could be due to the absence of TraI in the recipient cell.

DNA from the integrants obtained for both systems (pKK and pSU) was analysed by restriction analysis with an enzyme that only cuts the recipient *oriT*-containing plasmid. The results (**Figure 1B**) show that the proportion of cointegrate molecules co-residing with the unaltered recipient plasmid varies significantly depending on the system used. When a pMB1 replicon (pKK system) is used as recipient plasmid, there is roughly 50% of each molecular species, as previously shown [6]. However, when the p15A replicon (pSU system) is used, almost all molecules are cointegrates. This difference could reflect an involvement of the plasmid replication machinery in the reaction. Alternatively, since a similar amount of cointegrate molecules is found in both cases, we reasoned that this could be due to a minimal amount of cointegrate molecules required to resist the selection with Cm25. However, the same integration frequencies and proportion of cointegrate molecules were obtained when applying Cm selection of 10, 25 or 40 µg/ml (data not shown).

It was described that the DNA strand harbouring the *nic* site affected the efficiency of *oriT*-specific recombination mediated by TrwC [15]. When the *nic* site of the *oriT* was in the lagging strand, the percentage of recombined colonies increased 5 times; this was explained by the longer exposure of ssDNA in the lagging strand, which would favour TrwC nicking reaction. In both recipient plasmids used in the integration assays, the *nic* site was located in the lagging strand. We assayed two plasmids with the *nic* site in both orientations with respect to the replication fork, pLA58 (p220::*nic*▴), *nic* site in the lagging strand, and pLA59 (p220.2::*nic*▾), *nic* site in the leading strand. We mobilized the suicide plasmid to a recipient cell containing either pLA58 or pLA59 and no significant differences in the integration frequency obtained were observed (4.7×10^−6^ and 3.2×10^−6^ integrants per donor, respectively). Restriction analysis of the DNA of the integrants showed that the percentage of cointegrate molecules was around 50% when the recipient *nic* site was present in the lagging strand, and around 70–80% when the *nic* site was in the leading strand (**Figure 1C**). A plausible explanation is that TrwC is promoting the resolution of the cointegrate molecules preferentially when both *nic* sites lie on the lagging strand, as described in [15].

### Role of relaxosomal proteins TrwA and IHF in integration

The R388 relaxosome is formed by the *oriT* and proteins TrwC, TrwA, and host-encoded IHF. TrwA enhances the nicking activity mediated by TrwC on scDNA, while IHF acts as an inhibitor [7]. With respect to TrwC-mediated *oriT*-specific recombination on scDNA substrates, TrwA increases drastically the efficiency of the reaction in the absence of conjugation, and it also enhances TrwC-mediated site-specific recombination in the recipient cell by almost six times [15]. IHF was shown to inhibit recombination only in the absence of TrwA and on DNA substrates lacking one of the two TrwA and IHF binding sites [16]; in the presence of TrwA, however, the presence or absence of IHF was irrelevant.

To test the effect of TrwA and IHF in site-specific integration, the suicide plasmid (pR6K::*oriTp oriTw*) was mobilized to isogenic IHF+ or IHF− recipient strains containing the recipient plasmid pSU::*oriTw* in the presence or absence of a plasmid which expressed *trwA* (pET3a::*trwA*). The recipient plasmid pSU::*oriTp* was assayed as a negative control in all experimental conditions tested. The integration frequency was compared in wild type or IHF-deficient recipient strains in the presence or absence of TrwA. Results are shown in **Table 2**. Both TrwA and IHF are enhancers of the integration reaction, producing an increase of about 3.5 fold and more than 30 fold, respectively, in the integration frequency. DNA of the integrants was analyzed and the same restriction pattern was obtained from IHF− or IHF+ backgrounds (data not shown). The lack of both proteins in the recipient cell decreases by two logs the frequency of integration mediated by TrwC.

**Table 2:** Effect of TrwA and IHF on TrwC-mediated integration.

Recipient plasmid	IHF	TrwA	Integration frequency
pSU::*oriTw*	+	+	2.3×10^−4^
	+	−	6.5×10^−5^
	−	+	6.9×10^−6^
	−	−	1.6×10^−6^
pSU::*oriTp*	+	+	<9.4×10^−7^
	+	−	<9.4×10^−7^
	−	+	<7.9×10^−7^
	−	−	<7.9×10^−7^

Donor cells are II1 with pCIG1077 and pR6K::*oriT_P_ oriTw*, and recipient cells are DH5α (IHF+) or CIG1 (isogenic IHF−) containing the indicated recipient plasmid with (+TrwA) or without (−TrwA) plasmid pET3a:*trwA*. Data are the mean of three independent assays. Frequency is given in integrants per donor.

### Integration catalyzed by TrwC expressed from recipient cells

**Figure 2:**
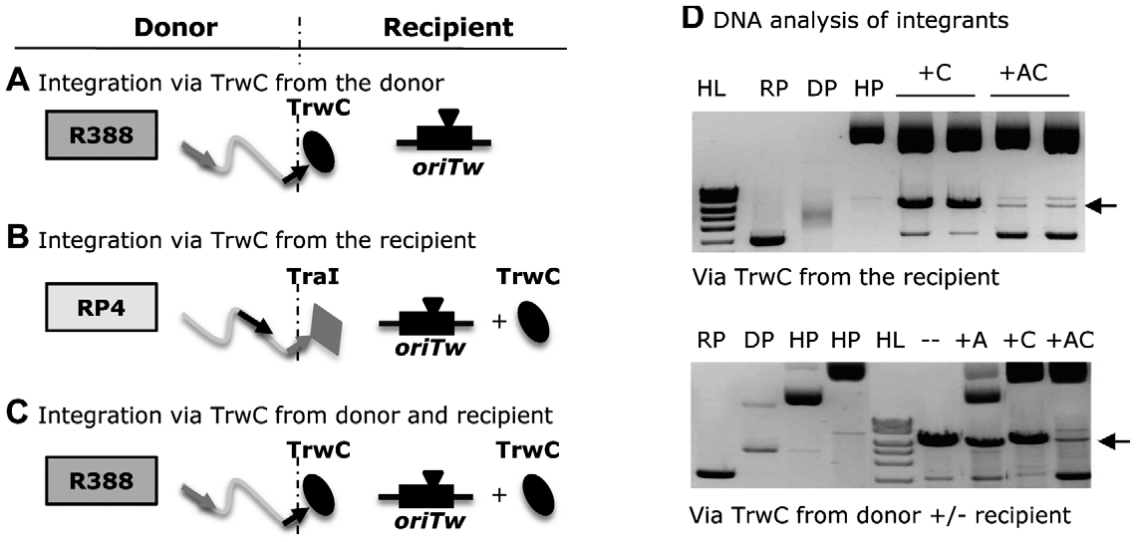
Integration assays expressing *trwC* from the donor and/or the recipient cell. **A**. Integration assay via TrwC from the donor. The suicide plasmid is mobilized by R388_TrwC relaxase from a II1 donor strain to a DH5α recipient which harbours plasmid pSU::*oriTw*. Integration mediated by TrwC takes place and the cointegrate is formed. **B**. Integration assay via TrwC from the recipient. The suicide plasmid is mobilized by RP4_TraI relaxase from an S17.1 λpir donor strain to a DH5α recipient which harbours pSU::*oriTw* and a plasmid coding for *trwC*. TrwC in the recipient is able to locate both *oriT*-containing plasmids and catalyze integration. **C**. Integration assay via TrwC from the donor and the recipient. The suicide plasmid is mobilized as in A. into a DH5α recipient which harbours plasmid pSU::*oriTw* plus a plasmid coding for *trwC*. R388 and RP4 non-mobilizable conjugative systems are represented with a dark and light grey square, respectively. TrwC is represented by an ellipse and RP4_TraI by a diamond. Arrowheads represent the *nic* site at the recipient *oriTw*. The T-strand of the suicide plasmid is represented by a wavy grey line containing the *oriTw* (black arrow) and the *oriTp* (grey arrow), and the recipient plasmid, with a thick black line. **D**. *XcmI* restriction pattern of integrant DNA. RP, recipient plasmid with R388 *oriT*; DP, suicide donor plasmid; HP, helper plasmid in the recipient. HL: Hyperladder MW marker. −, +A, +C, and +AC refer to the proteins produced by the helper plasmids in the recipient (none, TrwA, TrwC, or both). Top gel: integration assay via TrwC from the recipient (mobilization of donor plasmid with RP4-TraI). Bottom gel: integration assay via TrwC from the donor (mobilization of donor plasmid with R388-TrwC) with different helper plasmids in the recipient cell. The cointegrate molecular species is indicated with a black arrow.

The integration assay described (Figure 1A) is based on a conjugation assay, meaning that functional *oriT* and TrwC are required to achieve conjugative transfer of the suicide plasmid, which prevents the study of the DNA and protein requirements of the reaction. We assayed the ability of TrwC to catalyze site-specific integration when expressing *trwC* from the recipient cell as follows. The integration reaction mediated by TrwC also takes place upon the mobilization of the suicide plasmid to a recipient cell which contains a plasmid with an *oriT*, but in this case, the donor plasmid is mobilized by the RP4 conjugative system and *trwC* is being expressed from another plasmid in the recipient cell (**Figure 2**, compare A and B). When plasmid pSU1621 (pET3a:*trwC*) was present in the recipient, we observed an integration frequency of 1.4×10^−7^ (**Table 3**). Thus, *trwC* expressed in the recipient cell is able to locate both *oriT*-containing plasmids and catalyze integration. However, the frequency of this assay is 2–3 logs less than when we mobilized the suicide plasmid attached to TrwC from the donor cell (6.5×10^−5^ integrants per donor; Table 2).

**Table 3:** Integration assay expressing *trwC*+/−*trwA* in the recipient cell.

		*trwAC* expression in helper plasmid in the recipient				
Donor relaxase	Recipient plasmid	None	P*_T7_ trwC*	P*_trwA_ trwAC*	P*_tac_*− IPTG *trwC*	P*_tac_*+ IPTG *trwC*
RP4-TraI	pSU::*oriTw*	<2.2×10^−8^	1.4×10^−7^	4.2×10^−6^	6.0×10^−7^	3.0×10^−7^
RP4-TraI	pSU::*oriTp*	<2.2×10^−8^	<2.2×10^−8^	<2.2×10^−8^	<3.2×10^−8^	<3.2×10^−8^
R388-TrwC	pSU::*oriTw*	5.9×10^−5^	6.4×10^−6^	4.4×10^−6^	NT	NT

Donor cells harbouring the suicide mobilizable plasmid pR6K::*oriT_P_ oriTw* were either S17.1 λpir for TraI-mediated mobilization, or II1 (pCIG1077) for TrwC-mediated mobilization. Recipient strain was DH5α containing the indicated recipient and helper plasmids. The promoter driving the expression of *trwC* or *trwA+trwC* is indicated. The inducible Ptac promoter was assayed with (+IPTG) or without (−IPTG) 0,5 mM IPTG in the mating plate. Data are the mean of at least three independent assays. NT, not tested.

In an attempt to improve this frequency, we also expressed *trwA* in the recipient cell. The integration frequency increased by 30 fold (Table 3). Since the helper plasmid coding for *trwA+trwC* expresses these genes from the putative *trwABC* promoter of R388, the increase could be due to a higher expression level of *trwC* rather than to the presence of *trwA*. To examine this possibility, we expressed *trwC* in the recipient cell under the inducible *tac* promoter. The integration assay was performed on plates supplemented with IPTG to induce the expression of *trwC*. The results demonstrated that an increase in the production of TrwC in the recipient cell is not correlated with higher frequencies; in fact, a slight decrease in the integration frequency was observed (Table 3, last two columns), which could be due to toxicity of *trwC* overexpression. Thus, the improvement in the integration frequency when *trwC+trwA* are expressed in the recipient cell is due to the enhancing role of TrwA in the integration reaction.

DNA of the integrants was analyzed (Fig. 2D, top gel). The proportion of cointegrate molecules was much lower when TrwA is expressed in the recipient. So, TrwA, while enhancing TrwC-mediated integration, also promotes resolution of the cointegrates in the cell. Since *oriT*-specific recombination in the presence of both TrwA and TrwC is more efficient than integration, probably all molecules would be resolved if selection for the cointegrates was not applied.

Since TrwC-mediated site-specific integration can be obtained when TrwC is mobilized from the donor and also when *trwC* is expressed in the recipient, we reasoned that the integration frequency could be improved by joining both TrwC sources, as shown in Figure 2C. We mobilized the suicide plasmid by TrwC to a recipient harbouring the *oriT*-containing plasmid and a helper plasmid coding for *trwC*. The results, however, showed a decrease in the integration frequency of about 1-log compared to integration rates with no *trwC* expression in the recipient (Table 3, bottom row). When *trwA* was expressed together with *trwC* in the recipient, no recovery of this low frequency was observed (Table 3).

DNA analysis of integrants showed the same pattern observed in previous assays, with most molecules in the form of cointegrates except when TrwA is present in the recipient (Fig. 2D, bottom gel).

### TrwC requirements for integrase activity

The integration assay expressing *trwC* in the recipient cell allowed us to test TrwC mutants for their integrase activity. The following experiments were all performed mobilizing the suicide donor plasmid by the RP4 conjugative system.

**Figure 3:**
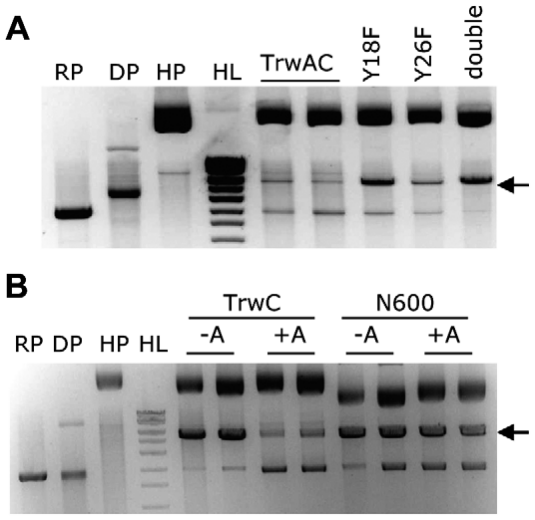
DNA analysis of colonies obtained in the integration assay from the recipient cell expressing different TrwC derivatives. **A**. TrwC catalytic mutants plus TrwA. **B**. TrwC wt or N600 with or without TrwA. Symbols as in Fig. 2.

We tested if the site-specific integration reaction requires the strand transferase activity of TrwC by using a set of point mutants in the catalytic tyrosyl residues: Y18F, Y26F, and the double mutant Y18FY26F. These mutants have been shown to affect conjugation and recombination reactions to different extents: Y18F strongly affects both activities, while Y26F has only a mild effect and the double mutant abolishes both processes [15]. We tested integration catalyzed by these TrwC mutants expressed from the recipient cell and no integrants were found, indicating that the reaction is dependent on both catalytic Tyr residues of the protein (**Table 4**). When TrwA was also produced in the recipient, a few integrants were obtained, allowing analysis of their DNA (**Figure 3A**). We observed that when Y18 was mutated, the resolution reaction was not favoured in spite of the presence of TrwA in the recipient, probably due to the fact that Y18 is the only tyrosine able to act on supercoiled DNA substrates. When only Y26 was mutated, although the integration reaction was reduced to residual levels, resolution of the cointegrates took place.

**Table 4:** TrwC requirements for site-specific integration.

Recipient plasmid	Helper plasmid in the recipient		Integration frequency
	TrwC	TrwA	
pSU::*oriTp*	wt	−	<6.6×10^−9^
pSU::*oriTw*	wt	−	1.3×10^−7^
	Y18F	−	<6.6×10^−9^
	Y26F	−	<6.6×10^−9^
	Y18F Y26F	−	<6.6×10^−9^
	N600	−	2.8×10^−8^
pSU::*oriTp*	wt	+	<2.9×10^−8^
pSU::*oriTw*	wt	+	4.2×10^−6^
	Y18F	+	6.6×10^−8^
	Y26F	+	3.0×10^−8^
	Y18F Y26F	+	4.0×10^−8^
	K502T	+	1.5×10^−6^
	N450	+	3.4×10^−8^
	N600	+	2.8×10^−7^

Integration frequencies (integrants per donor) obtained when expressing in the recipient cell the indicated *trwC* derivatives, in the presence or absence of TrwA. Donor cells are S17.1 λpir with pR6K::*oriT_P_ oriTw* and recipient cells DH5α with the indicated recipient and helper plasmids. Data are the mean of at least four independent assays.

The C-terminal DNA helicase domain of TrwC is required for conjugative DNA transfer, as it supports unwinding of the DNA to be transferred. Its involvement in the integration reaction was tested using a TrwC point mutant (K502T) affecting motif I of the helicase superfamily I (Walker A box, GxG**K**T); this mutant is deficient in conjugation and proficient in recombination [15]. TrwC(K502T) showed a decrease of less than 3 fold in the integration frequency when compared to TrwC wt (Table 4). A similar slight decrease was observed in the recombination assay, which was attributed to the observation that TrwC(K502T) showed roughly four times less protein product when compared to wild type in a western blot [15]. Thus, the DNA helicase activity is dispensable for TrwC-mediated site-specific integration.

The minimal domain of TrwC able to promote site-specific recombination efficiently was TrwC-N600 [15]. To delimit the minimal TrwC domain sufficient to catalyze integration, we assayed N600 and N450 expressed in the recipient together with TrwA (Table 4). N450 catalyzes integration with about 2-log lower efficiency than wild-type while N600 catalyzes integration about ten times less efficiently than full-length TrwC. This efficiency decreased in about 1-log when TrwA was not expressed in the recipient (Table 4), meaning that TrwA is helping this domain as it helps TrwC-mediated integration. DNA of the integrants was analysed by restriction analysis (**Figure 3B**). In contrast to full-length TrwC, TrwA does not promote the resolution of the cointegrates formed by N600. This result suggests that TrwA can play different roles in integration (enhancing the reaction catalyzed by both TrwC and N600) and in recombination (promoting TrwC-mediated, but not N600-mediated resolution of the cointegrates).

### *oriT* requirements for integration

**Figure 4:**
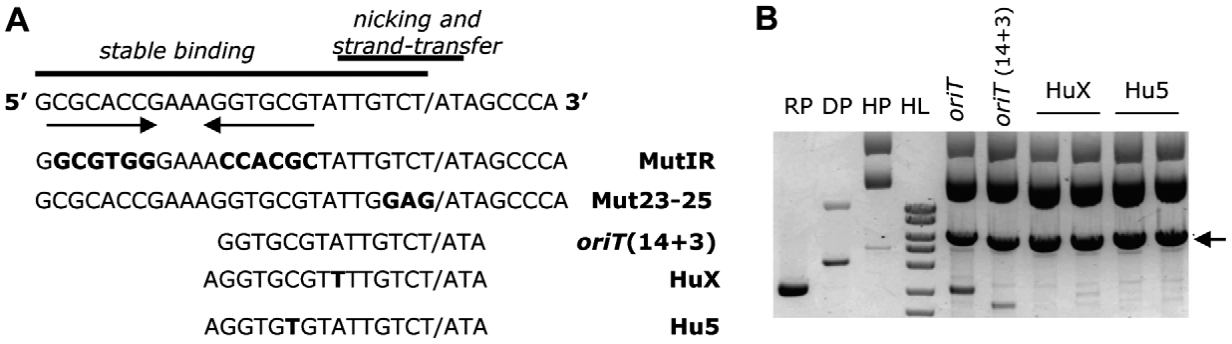
Integration assays on different target sequences. **A**. DNA sequence of the central R388 *oriT* region, coordinates 201 to 169 from [18]. The arrows show inverted repeat IR2. The *nic* site is indicated by a slash. Horizontal bars indicate minimal sequence requirements for different TrwC activities. Below are shown the DNA sequences of the *oriT* mutations MutIR and Mut23-25, the minimal *oriTw* (*oriT* 14+3), and the human sequences resembling the R388 *nic* site, HuX and Hu5. **B**. DNA analysis of integrants obtained with recipient plasmids containing the indicated target sequences. Symbols as in Fig. 2. RP containing the minimal *oriT* is around 300 bp shorter than RP with full-length *oriT*.

We have analyzed the specificity of TrwC for its integration target, the R388 *oriT* (*oriTw*). The nicking and binding sites of TrwC (**Figure 4A**) are the minimal *oriTw* requirements for different TrwC activities *in vivo* and *in vitro* (see Introduction). To test the requirement of the binding site we have assayed the mutIR *oriT*, in which the sequence of both arms of the inverted repeat IR2 to which TrwC binds was changed by another sequence maintaining the secondary structure (Fig. 4A). This mutation provokes a drastic 5-log reduction of the mobilization frequency [8]. To test the requirement of the nicking site, we used mut23-25 *oriT* (Figure 4A), which has 3 nucleotides changed 5′ to the *nic* site. This mutation almost abolishes mobilization, as this region is critical for the initial nicking reaction [8]. We tested TrwC-mediated integration with recipient plasmids carrying those mutations and expressing *trwC* and *trwA* in the recipient cell, and we did not detect any integration event in any case (integration frequency <10^−8^ integrants per donor), confirming that TrwC also requires its binding and nicking sites to catalyze integration. The same mutations were tested in the integration via TrwC from the donor, i.e. when TrwC enters the recipient cell covalently attached to the transferred DNA strand. The results are shown in **Table 5**. In this case, only a modest decrease (5 to 9-fold) in the integration frequency was observed, indicating different TrwC DNA requirements at the initiation and termination steps of the integration reaction (see Discussion).

**Table 5:** DNA targets for TrwC-mediated integration.

Recipient plasmid	TrwA	Integration frequency
pSU::*oriTp*	+	<3.3×10^−8^
pSU::*oriTw*	+	2.3×10^−4^
pSU::*oriTw* (mut IR)	−	1.3×10^−5^
pSU::*oriTw* (mut 23-25)	−	7.3×10^−6^
pSU::*oriTw* (14+3)	−	7.4×10^−7^
pSU::*oriT* HuX 15+3*(−7)*	+	5.3×10^−7^
pSU::*oriT* Hu5 15+3*(−10)*	+	4.6×10^−7^

Donor cells are II1 with pCIG1077 and pR6K::*oriT_P_ oriTw*, and recipient cells are DH5α containing the indicated recipient plasmid, with (+TrwA) or without (−TrwA) plasmid pET3a::*trwA*. Data are the mean of at least three independent assays. Frequency is given in integrants per donor.

In the recombination reaction mediated by TrwC, it was found that one of the *oriT* target copies could be reduced to 17 bp (14+3) containing the *nic* site and the proximal arm of the IR2, maintaining a recombination frequency of 80% [15]. To test if TrwC was also able to promote integration into a recipient plasmid containing this minimal *oriT*, plasmid pSU::*oriT*(14+3) (coordinates 174 to 190 from ref. [18]) was used as recipient plasmid (Fig. 4A). The results (Table 5) show that TrwC is able to promote site-specific integration on this minimal *oriT*; however, the reaction decreases by around 2 logs. When *trwA* was also expressed in the recipient cell, a modest increase in integration frequency was obtained (Table 5). DNA analysis of the integrants showed the same proportion of cointegrates when the recipient plasmid carried full-length or minimal *oriT* (**Figure 4B**).

In a recent report, we demonstrated that TrwC can promote site-specific recombination on substrate plasmids where the *oriT1* copy was replaced by human genomic DNA which contained sequences resembling the essential *oriT* core region (Fig. 4A): two (15+3) sequences, located in chromosomes 5 and X, with no mismatches in the essential core sequence (6+2), worked as targets with the same efficiency as with the canonical 14+3 sequence [17]. We assayed TrwC mediated site-specific integration into recipient plasmids containing these human targets (HuX 15+3(−7) and Hu5 15+3(−10); Fig. 4A) and a helper plasmid which provided TrwA. TrwC catalyzed site-specific integration into both human target sequences; the efficiency of the reaction decreases by only 2–3 times compared to the minimal *oriT*(14+3) (Table 5). When DNA from the integrants was analyzed, we found that 100% of the molecules were cointegrates (Fig. 4B). This suggests that the deviations from the consensus in the human sequences, lying out of the essential nicking region, do not affect significantly termination of the integration reaction, but prevent resolution of the cointegrates formed.

### Integration into chromosomal DNA

**Figure 5:**
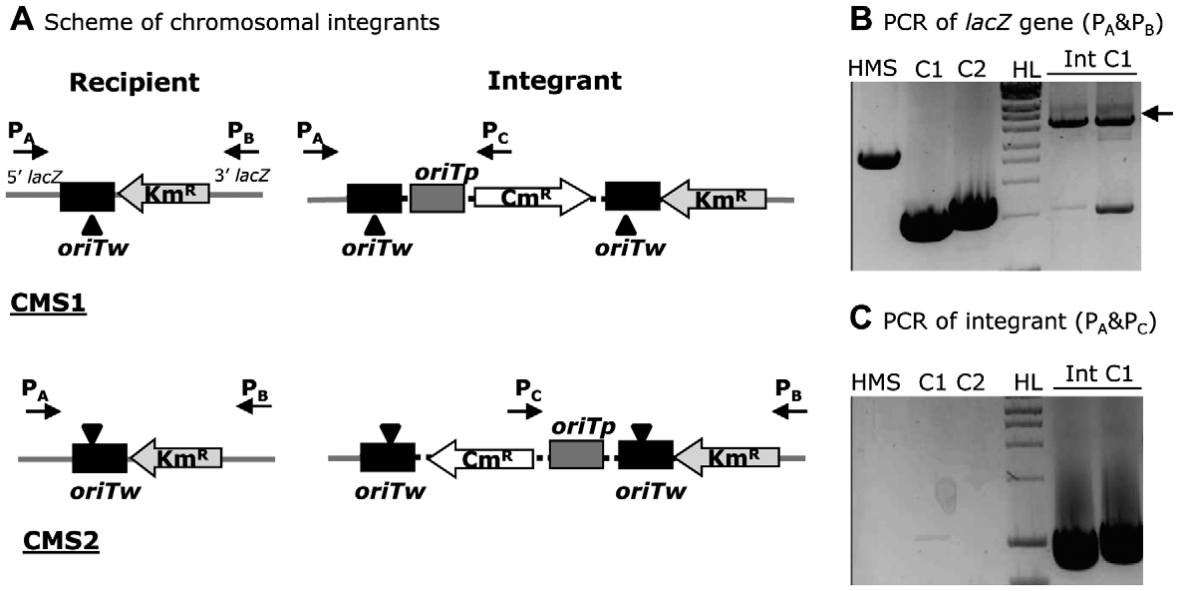
Integration assay in the bacterial chromosome. **A**. Scheme of the expected integrants in the chromosomal *oriT* copy of recipient strains CMS1 or CMS2. Symbols as in Fig. 1. **B and C**. PCR amplification with oligonucleotides P_A_ and P_B_, flanking the *lacZ* gene (in B), or with P_A_ and P_C_, annealing only to the cointegrate (in C). C1, C2 and HMS, strains CMS1, CMS2, and HMS174, used as a negative control. Int C1, integrants obtained with recipient strain CMS1. The black arrow indicates the amplification product of the suicide plasmid integrated in the chromosomal *oriT* copy. HL: MW marker.

We tested the ability of TrwC to catalyze site-specific integration into a chromosomal *oriT* copy. In the *E.coli* chromosome we did not find any sequences which resemble the minimal R388 *oriT* sequence required for TrwC binding and nicking. Two recipient strains were constructed containing an *oriTw* copy plus a resistance marker in place of the chromosomal *lacZ* gene (see Experimental Procedures): CMS1 and CMS2, with the *oriTw nic* site lying in the lagging and leading strands with respect to the replication fork, respectively (**Figure 5A**). The integration assay was performed by mobilizing the suicide plasmid (pR6K::*oriTp oriTw*) either with R388-TrwC or with RP4-TraI to recipient strains CMS1 or CMS2, or HMS174 (the isogenic *oriT*- strain) as a negative control. We did not obtain any integrant in any case (<10^−8^ integrants per donor) except when mobilizing the suicide plasmid with TrwC and using CMS1 as recipient. The integration frequency was low (1.1×10^−7^ integrants per donor, mean of five independent assays) but the results were reproducible. DNA analysis of the integrants was done by PCR amplification of the *lacZ* gene (**Figure 5B**). Amplification products of the expected size were obtained in all cases: 3 kb (size of the *lacZ* gene) in HMS174, 1.5 kb in CMS1 and CMS2 (size of the *oriT*-Km cassette in place of the *lacZ* ORF), and around 5 kb for the integrants (which contain the integrated suicide plasmid). It can also be observed that a 1.5 kb product is also visible, indicating that the integration reaction is reversible. In addition, specific amplification of a region of the cointegrate was performed with primers P_A_ and P_C_ (**Figure 5C**). This specific 850 bp amplification product was gel-extracted and the DNA sequence was determined, confirming the expected integration structure.

The negative controls (*oriT*- recipient strain, or mobilization with RP4-TraI to any of the strains: <4.3×10^−8^ integrants per donor in all cases) indicate that the reaction is dependent on TrwC and its target R388 *oriT*. Thus, TrwC is able to catalyze the integration reaction into the chromosome although with 2–3 logs lower efficiency than into an *oriTw*-containing plasmid. Interestingly, no integration events were detected when using CMS2 as a recipient strain. This is likely due to the fact that the *nic* site of the *oriTw* lies in the leading strand of replication, meaning that it could be less accessible to TrwC. So, the exposure of single-stranded DNA, which was shown not to be a requisite in integration into an *oriTw*-containing plasmid, is a relevant factor in order to obtain integration into the host chromosome; in plasmid DNA, this exposure may be easier to obtain by local supercoiling than in the chromosome.

## Discussion

The conjugative relaxase TrwC is the protein in charge of piloting the transferred DNA into the recipient cell during bacterial conjugation of plasmid R388. It was reported that this protein acts not only as a conjugative relaxase, but also as a site-specific recombinase and integrase in recipient bacteria [6]. By modifying the donor bacterial strain and the recipient *oriT*-containing plasmid, we have significantly improved the integration frequency reported previously (Table 1). Using different types of integration assays (Fig. 2), we have tried to elucidate the underlying mechanism of the site-specific integration reaction mediated by TrwC in bacteria. Taken together, the results obtained suggest that the reactions mediated by TrwC to accomplish integration mimic those for initiation and termination of conjugation; this assumption is based on the following evidences:

- TrwC-mediated site-specific integration was favoured by the presence of the R388 relaxosome components IHF and TrwA in the recipient cell (Table 2). This contrasts with TrwC-mediated site-specific recombination tested on plasmids containing two *oriT* copies, where the observed effect of TrwA (enhancer) and IHF (null or repressor under certain conditions) correlated with their described effect on TrwC nicking on supercoiled DNA. This difference probably reflects the fact that recombination is boosted by two initial nicking reactions, while integration in the recipient requires a final strand-transfer reaction. Thus, while a complete relaxosomal complex may be a reluctant substrate for nicking by TrwC, it behaves as an optimal substrate for strand transfer by a TrwC-DNA complex.
- We have shown that the integration reaction also took place when *trwC* was expressed only in the recipient, which implies that TrwC is able to locate both targets to perform strand transfer reactions. Curiously, when TrwC-DNA entered from the donor cell, additional expression of *trwC* in the recipient caused a decrease in the integration frequency (Table 3). This could be likely due to the fact that free TrwC molecules are bound to the *oriT* of the recipient plasmid, preventing strand transfer from incoming TrwC-DNA complexes.
- A double mutant in both TrwC catalytic residues (Y18FY26F) was inactive, confirming that nicking and strand transfer reactions are required for conjugation, recombination and integration. However, integration was totally dependent on both catalytic residues of TrwC, Y18 and Y26 (Table 4), while the Y26F mutation is well tolerated in recombination [15]. Y18 and Y26 are proposed to act in the initiation and termination of conjugative DNA transfer [12], [19]; it has been proposed that Y26 could have access to the *nic* site on supercoiled DNA once Y18 has formed the covalent complex [19]. Thus, while recombination requires two initiation nicking reactions both catalyzed by Y18, integration requires initiation and termination events.
- It has been described that formation of the IR2 hairpin at *oriT* enhances Y18 activity [19]. It can be expected that TrwC requirements for *oriT* recognition are more strict for the initial nicking reaction than for strand-transfer into a second target once the covalent complex is formed with the first target. This model correlates with our observation that changes in the critical area affecting either the *nic* or the binding sites abolished TrwC integration activity when TrwC was only expressed in the recipient cell and thus, it is required to act on the supercoiled mutant *oriT*. In contrast, incoming TrwC-DNA complexes could transfer DNA into acceptor sites with mismatches in the core *oriT* sequence (Table 5).

**Figure 6:**
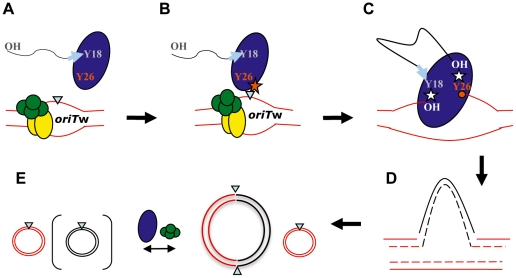
A model for TrwC-mediated site-specific integration. **A**. TrwC (blue oval) arrives to the recipient cell covalently bound to the suicide plasmid (dashed line) through the Y18 residue. The recipient contains the recipient plasmid (red lines). TrwA (green spheres) and IHF (yellow ovals) sit on the *oriTw* forming a relaxosome conformation which increase the exposure of ssDNA and the *nic* site (blue triangle). **B**. The incoming TrwC-DNA complex has a free Y26 residue. Y26 nicks (orange star) and binds covalently to the recipient *nic* site. **C**. Strand-transfer reactions are produced by the attack of the free –OH groups generated to Y18 covalently bound to the suicide plasmid and to Y26 attached to the recipient plasmid. As a result, the transferred DNA strand is integrated into the recipient plasmid. **D**. The host replication machinery duplicates the integrated DNA. **E**. Two molecules are obtained, the cointegrate and the recipient plasmid (*nic* sites indicated by triangles). The cointegrate can be resolved by TrwC-mediated site-specific recombination (enhanced by TrwA), producing the two initial molecules. The suicide plasmid is lost, since it cannot be replicated in the recipient cell.

**Figure 6** depicts a model for the integration reaction based on the above mentioned results. A TrwC-DNA complex enters the recipient cell, where a supercoiled *oriT*-containing acceptor plasmid is present. TrwC is attached to the T-strand through Y18, the residue responsible for the initial nicking event on supercoiled DNA (Fig. 6A). The relaxosome formed at the recipient *oriT* with TrwA and IHF would be the preferred substrate for the free Y26 residue in the TrwC-(Y18)-DNA complex, which nicks the recipient *oriT* and forms a covalent intermediate (Fig. 6B); free TrwC molecules, however, would sit on the *oriT* copy and inhibit integration of incoming TrwC-DNA complexes. In this intermediate, there are two free 3′-OH ends which could attack each of the covalent Y-DNA complexes; as a result, the T-DNA strand would be integrated into the recipient *oriT* (Fig. 6C). Integration requires an additional strand-transfer reaction compared to conjugative DNA transfer, which may explain why the Y26F mutation affects integration more than conjugation. The resolution of the covalent intermediate (Fig. 6D) is probably mediated by the host replication machinery, as previously suggested for site-specific recombination [15]. Finally, in the presence of TrwC and TrwA, the cointegrate molecules would be resolved (Fig. 6E), giving rise to a higher proportion of recipient plasmids, as observed (Figs. 2, 3). This model is based on the action of a single TrwC monomer, as previously suggested also for conjugation [19], since the existence of two catalytic Tyr residues allows a monomer to perform both strand-transfer reactions; however, previous evidences of TrwC oligomerization *in vitro* [20] and DNA-independent TrwC transfer into recipients [6] leave open the possibility that TrwC acts as an oligomer both in donor and recipient cells.

Only a few relaxases have been reported to catalyze site-specific recombination, and the only other conjugative relaxase reported to catalyze intermolecular recombination is that of the self-transfer system of the integrative and conjugative element ICE*clc* [21]. This difference among conjugative relaxases has no correlation with taxonomic proximity (according to [22]), or with the number of catalytic Tyr residues. Intriguingly, the relaxase of ICE*clc* can act on two different *oriTs* for ICE transfer with similar efficiencies; however, it is able to catalyze *in vivo* strand-exchange between two plasmids carrying *oriT1* but not when carrying *oriT2* [21]. This result points to differences in the interaction of the relaxase with other relaxosomal proteins or with the *oriT* as factors which could determine the recombinase/integrase activity of relaxases, rather than to intrinsic catalytic differences among these proteins.

No other relaxase has been reported to perform site-specific integration of incoming DNA in recipient bacteria. In addition, we show here that TrwC could integrate the incoming DNA into a chromosomal *oriT* copy (Figure 5). Considering that R388 is a broad host range plasmid, the ability of TrwC to integrate the incoming DNA strand into a recipient chromosomal target may allow integration of R388 into the genome of non-permissive recipient hosts if a suitable target sequence exists in the recipient genome. We have shown that the 17 bp core of the R388 *oriT* was sufficient to act as a target for TrwC-mediated integration, and that DNA requirements are less stringent on the acceptor target site, broadening the possibility of finding natural TrwC targets in any recipient genome. This would confer R388 the possibility to colonize a broader range of microbial hosts in nature.

There have been reports of conjugative DNA transfer into different types of eukaryotic cells, implying that TrwC bound to a DNA molecule of any length could also have access to eukaryotic genomes. In this work, we show that TrwC catalyzed integration into two DNA sequences found in the human genome which resemble the minimal *oriT* (Table 5). Interestingly, integrants formed at these sites did not revert (Fig. 4B), so integration events obtained in this way would be stable, overcoming the limitation of reversible recombinases such as Cre [23]. Our results, together with previous reports on TrwC targeting to the nucleus [17], underscore the potential of TrwC as an integrase for genomic engineering of higher organisms.

## Materials and Methods

### Bacterial strains and growth conditions

*E. coli* strains II1 [24] and S17.1 λpir [25] were used as donor cells for the integration assays. As recipients, the following *E. coli* strains were used, as indicated: DH5α λpir [26], as a conjugation control of the suicide plasmid; DH5α [27] and CIG1 [16], as isogenic IHF+ and IHF− strains; and HMS174 [28], CMS1 and CMS2 (this work, see below), as isogenic strains without or with a chromosomal *oriT* copy, in both orientations. Bacteria were grown in Luria-Bertani (LB) broth, supplemented with agar for solid culture. For selection, antibiotics were used at the following concentrations: ampicillin (Ap), 100 µg/ml; chloramphenicol (Cm), 25 µg/ml; erythromycin (Em), 200 µg/ml; kanamycin (Km), 25 µg/ml; nalidixic acid (Nx), 20 µg/ml; rifampin (Rif), 100 µg/ml; streptomycin (Sm), 300 µg/ml. Thymidine (dT) was supplemented when necessary to a final concentration of 0.3 mM. IPTG was added in LB plates to a final concentration of 0.5 mM.

### Plasmids and plasmid constructions

Published plasmids used in this work are listed in **Table S1**, and plasmids constructed for this work are detailed in **Table S2**. Plasmids were constructed using standard cloning procedures [29]. Restriction enzymes, Shrimp Alkaline Phosphatase, and T4 DNA ligase were purchased form Fermentas. For PCR amplification, high fidelity Vent DNA polymerase (New England BioLabs) was used. Primers used for PCR were obtained from Sigma-Aldrich. DNA sequences of all cloned PCR segments were determined.

### Strain constructions

CMS1 and CMS2 are isogenic strains carrying an R388 *oriT* copy and a kanamycin resistance marker in place of the chromosomal *lacZ* ORF of *E. coli* strain HMS174. These strains were used as recipients to assay integration into the chromosome. The *oriT-km^R^* cassette was introduced in the chromosome by the method of Dantsenko and Wanner [30] disrupting the *lacZ* gene. Oligonucleotides used for PCR reactions were 5′-ATGACCATGATTACGGATTCACTGGCCGTCGTTTTACAACACAGCTATGACCATGATTAC-3′, containing 40 bases of the 5′ region of the *lacZ* gene and annealing towards the *oriT*, and 5′-GACACCAGACCAACTGGTAATGGTAGCGACCGGCGCCAGCTGCTAAAGGAAGCGGAACA-3′, with 40 bases homologous to the 3′ end of the *lacZ* gene and annealing towards the *nptII* gene. Template DNAs were pCMS17 (*oriTw*▴-Km) and pCMS18 (*oriTw*▾-Km) (Table S2). One hundred nanograms of each PCR product were transformed into arabinose-induced HMS174 cells harbouring plasmid pKD20, coding for an L-arabinose-inducible λRed recombinase. Transformants were grown at 30°C and plated on LB+Km. Km resistant colonies were confirmed by their white colour on plates supplemented with X-gal and by PCR analysis using oligonucleotides P_A_ (5′-ATGACCATGATTACGGATTCA-‘3) and P_B_ (5′-GACACCAGACCAACTGGT-‘3), annealing to the 5′and 3′ ends of the *lacZ* gene, respectively.

### Mating assays

Standard mating assays were performed as described [12], incubating the mating mixture for 4 hours at 37°C. Conjugation frequencies are expressed as number of transconjugants per donor cell.

### Integration assays

TrwC-mediated site-specific integration assay described in [6] was optimized as follows. Matings were done using II1 as donor strain containing a plasmid harbouring an *oriTw*, an *oriTp* and an R6K replicon (only replicates in strains expressing *pir*), used as a suicide plasmid for mobilization into strains lacking the Pir protein. The suicide plasmid was mobilized from II1 by a non mobilizable plasmid (pCIG1077) coding for the transfer region of plasmid R388 except for the *oriT*. DH5α was used as a recipient harbouring a plasmid with R388 *oriT* (*oriTw*) or RP4 *oriT* (*oriTp*), as a negative control. Integrants were selected in Cm plates, as it is the resistance provided by the suicide plasmid upon integration. The frequency is reported as the number of integrants per number of donor cells. Conjugative transfer controls were performed in all experiments using *pir* strains as recipients, and transfer efficiency was always close to 100% transconjugants/donor. As a negative control for integration, the suicide plasmid (containing *oriTp* and *oriTw*) was mobilized from S17.1 λpir by RP4_TraI to the same recipient as in the test. Thus, TrwC is not present in the reaction, but the *oriTw* enters in a single-stranded form, which allow us to rule out other causes of recombination in the recipient cell. In the integration assay via TrwC from the recipient, *trwA* and/or *trwC* were expressed in recipient cells under the control of the T7 promoter, to avoid possible toxic effects of overexpression.

Integration events were also analysed at the molecular level. Plasmid DNA was analysed by restriction analysis with enzymes which cut only once in the recipient plasmid and do not cut the donor plasmid (*BstEII*, *NdeI* or *XcmI*, as appropriate), to estimate the proportion of cointegrates. For PCR analysis, DNA was isolated from the colonies with Instagene (Bio-Rad). Primers P1 (5′-AGCGGATAACAATTTCACACAGGA-3′), annealing to the recipient plasmid, and P2 (5′-GCAGGATCCGCTAAGCTTTGTCGGTCATTTCGA-3′), annealing to the donor plasmid downstream *oriTw*, were used to obtain an amplicon with a size of 1.2 kb only expected for the cointegrate molecules. As a positive control we used pKK::*oriT-Km* [6], which contains 826 bp of the suicide plasmid, mimicking a possible cointegrate molecule.

The analysis of the integrants obtained by TrwC-mediated site-specific integration into the chromosome were analyzed as follows. When using CMS1 as recipient strain, primers P_A_ and P_C_ (5′-GCCTCAAAATGTTCTTTACGA-3′), annealing to 3′ end of the *cat* gene, were used to amplify a specific region (850 bp) of the integrant. To verify integrants obtained when using CMS2 as recipient cell, primers P_C_ and P_B_ were used to amplify a band of 1800 bp specific of this type of integrant.

## Supporting Information

Table S1 **Published plasmids used in this work.**
(DOCX)

Table S2 **Plasmids constructed for this work.**
(DOCX)
