# Supplementary material for: Site-Specific Integration of Foreign DNA into Minimal Bacterial and Human Target Sequences Mediated by a Conjugative Relaxase
Source: PLoS One. 2012 Jan 23;7(1):e31047. doi: 10.1371/journal.pone.0031047 (PMC3264647; doi:10.1371/journal.pone.0031047)
Supplement: Table S1 — Published plasmids used in this work. (DOCX) [file pone.0031047.s001.docx]

**Table S1. Published plasmids used in this work**

| **Plasmid** | **Ab^R^** | **Description** | **Reference** |
| --- | --- | --- | --- |
|  |  |  |  |
| p220.2 | Ap^R^, Hyg^R^ | EBV-based shuttle vector | [1] |
| pCIG1003 | Ap^R^ | pET3a::*trwAC*(Y18F) | [2] |
| pCIG1010 | Ap^R^ | pET3a::*trwAC*(Y26F) | [2] |
| pCIG1026 | Ap^R^ | pET3a::*trwAC*(K502T) | [2] |
| pCIG1028 | Cm^R^ Km^R^ | *oriT* recombination substrate | [2] |
| pCIG1051 | Ap^R^ | pET3a::*trwAC*(N450) | [2] |
| pCIG1057 | Cm^R^ Km^R^ | As pCIG1028, inverted *oriT1* | [2] |
| pCIG1077 | Ap^R^ | pKK223-3::*P_ABC_trwA-trwL* | [3] |
| pCIG1079 | Cm^R^ Km^R^ | As pCIG1028, *oriT1*(174-190), *oriT2*(1-191) | [2] |
| pCIG1089 | Ap^R^ | pET3a::*trwAC*(N600) | [2] |
| pCIG1099 | Ap^R^ | pET3a::*trwC*(N600) | [2] |
| pCIG1116 | Cm^R^ Km^R^ | As pCIG1028, *oriT1* HuX 15+3*(-7)* | [4] |
| pCIG1117 | Cm^R^ Km^R^ | As pCIG1028, *oriT1* Hu5 15+3*(-10)* | [4] |
| pET3::*trwA* | Ap^R^ | pET3a::*trwA* | [2] |
| pET3::*trwAC* | Ap^R^ | pET3a::*P_trwA_trwAC* | [5] |
| pET3::*trwAC*mut | Ap^R^ | pET3a::*P_trwA_trwAC*(Y18FY26F) | [5] |
| pET29::*trwAC* | Km^R^ | pET29c::*P_trwA_trwAC* | [5] |
| pKD20 | Ap^R^ | Red recombinase expression | [6] |
| pKK223-3 | Ap^R^ | Expression vector | Pharmacia |
| pKK::*oriT* | Ap^R^ | pKK223-3:: *oriT* | [5] |
| pKK::*oriT-Km* | Ap^R^, Km^R^ | pKK223-3::*oriT+ nptII* | [5] |
| pR6K::*oriT_P_ oriTw* | Cm^R^ | oriV(R6K):: *oriT_p_+oriT_w_* | [5] |
| pSU36 | Km^R^ | Cloning vector | [7] |
| pSU39 | Km^R^ | Cloning vector | [7] |
| pSU1186 | Ap^R^ | pUC8::*oriT* | [8] |
| pSU1483  pSU1600 | Ap^R^  Ap^R^ | pKK223-3::*trwC*  pET3a::*trwC*(N293) | [9]  [2] |
| pSU1621 | Ap^R^ | pET3a::*trwC* | [10] |
| pSU1654 | Ap^R^ | pET3a::*trwC*(Y18F) | [10] |
| pSU1655 | Ap^R^ | pET3a::*trwC*(Y18FY26F) | [10] |
| pSU1659 | Ap^R^ | pET3a::*trwC*(Y26F) | [10] |
| pSU1673 | Cm^R^ | pUC18::*oriTw*(mut23-25) | [10] |
| pSU1678 | Cm^R^ | pUC18::*oriTw*(mutIR) | [11] |
| pSU2007 | Km^R^, Tp^R^ | R388 Km^R^ | [12] |
|  |  |  |  |

**References**

1. Yates JL, Warren N, Sugden B (1985) Stable replication of plasmids derived from Epstein-Barr virus in various mammalian cells. Nature 313: 812-815.

2. César CE, Machón C, de la Cruz F, Llosa M (2006) A new domain of conjugative relaxase TrwC responsible for efficient oriT-specific recombination on minimal target sequences. Mol Microbiol 62: 984-996.

3. César CE, Llosa M (2007) TrwC-mediated site-specific recombination is controlled by host factors altering local DNA topology. J Bacteriol 189: 9037-9043.

4. Agúndez L, Machón C, César CE, Rosa-Garrido M, Delgado MD, et al. (2011) Nuclear Targeting of a Bacterial Integrase That Mediates Site-Specific Recombination between Bacterial and Human Target Sequences. Appl Environ Microbiol 77: 201-210.

5. Draper O, Cesar CE, Machon C, de la Cruz F, Llosa M (2005) Site-specific recombinase and integrase activities of a conjugative relaxase in recipient cells. Proc Natl Acad Sci U S A 102: 16385-16390.

6. Datsenko KA, Wanner BL (2000) One-step inactivation of chromosomal genes in Escherichia coli K-12 using PCR products. Proc Natl Acad Sci U S A 97: 6640-6645.

7. Bartolomé B, Jubete Y, Martínez E, de la Cruz F (1991) Construction and properties of a family of pACYC184-derived cloning vectors compatible with pBR322 and its derivatives. Gene 102: 75-78.

8. Llosa M, Bolland S, de la Cruz F (1991) Structural and functional analysis of the origin of conjugal transfer of the broad-host-range IncW plasmid R388 and comparison with the related IncN plasmid R46. Mol Gen Genet 226: 473-483.

9. Grandoso G, Llosa M, Zabala JC, de la Cruz F (1994) Purification and biochemical characterization of TrwC, the helicase involved in plasmid R388 conjugal DNA transfer. Eur J Biochem 226: 403-412.

10. Guasch A, Lucas M, Moncalián G, Cabezas M, Pérez-Luque R, et al. (2003) Recognition and processing of the origin of transfer DNA by conjugative relaxase TrwC. Nat Struct Biol 10: 1002-1010.

11. Lucas M, Gonzalez-Perez B, Cabezas M, Moncalian G, Rivas G, et al. (2010) Relaxase DNA binding and cleavage are two distinguishable steps in conjugative DNA processing that involve different sequence elements of the nic site. J Biol Chem 285: 8918-8926.

12. Martínez E, de la Cruz F (1988) Transposon Tn21 encodes a RecA-independent site-specific integration system. Mol Gen Genet 211: 320-325.
