# Supplementary material for: Site-Specific Integration of Foreign DNA into Minimal Bacterial and Human Target Sequences Mediated by a Conjugative Relaxase
Source: PLoS One. 2012 Jan 23;7(1):e31047. doi: 10.1371/journal.pone.0031047 (PMC3264647; doi:10.1371/journal.pone.0031047)
Supplement: Table S2 — Plasmids constructed for this work. (DOCX) [file pone.0031047.s002.docx]

**Table S2. Plasmids constructed for this work**

|  |  | **Construction (1)** | | |
| --- | --- | --- | --- | --- |
| **Plasmid** | **Description** | **Vector** | **Insert** | **Digestion** |
| pCMS17 | As pCIG1057 *ΔlacI^q^* | pCIG1057 | --- | *XbaI*/religation |
| pCMS18 | As pCIG1028 *ΔlacI^q^* | pCIG1028 | --- | *XbaI*/religation |
| pLA30 | pKK223-3::*oriTp* | pKK223-3 | RP4 | *EcoRI*-*HindIII* CCAAGAATTCCGCTTGCCCTCATCTG  CCAAAAGCTTGCGCTTTTCCGCTGCATAA |
| pLA31 | pSU36::*oriTp* | pSU36 | pLA30 | *EcoRI*-*HindIII* |
| pLA32 | pSU36::*oriTw* | pSU36 | pSU1186 | *EcoRI*-*HindIII* |
| pLA33 | pSU36::*oriTw*(mutIR) | pSU36 | pSU1678 | *EcoRI*-*HindIII* |
| pLA34 | pSU36::*oriTw*(mut23-25) | pSU36 | pSU1673 | *EcoRI*-*HindIII* |
| pLA48 | pSU39::*oriT* HuX 15+3*(-7)***(2)** | pSU39 | pCIG1116 | *HindIII*-*XbaI* |
| pLA49 | pSU39::*oriT* Hu5 15+3*(-10)***(2)** | pSU39 | pCIG1117 | *HindIII*-*XbaI* |
| pLA51 | pSU39::*oriT*(14+3)**(3)** | pSU39 | pCIG1079 | *HindIII*-*XbaI* |
| pLA58 | p220.2::*oriT▲* (*nic* antisense) | p220.2 | PCR on pSU2007 | *XbaI*-*HindIII*  CCATCTAGACTCATTTTCTGCATCATTGT  AACAAGCTTCCTCTCCCGTAGTGTTAC |
| pLA59 | p220.2::*oriT▼* (*nic* sense) | p220.2 | PCR on pSU2007 | *XbaI*-*HindIII*  CCATCTAGACCTCTCCCGTAGTGTTAC  CCAAAGCTTCTCATTTTCTGCATCATTGT |

**(1)** First column lists the vector plasmids; second column lists the plasmids from which the inserts were obtained, and third column indicates either the restriction enzymes used for cloning, or the oligonucleotides used for PCR amplification of the desired fragment, with the restriction sites underlined.

**(2)** Human sequences in the indicated human chromosomes (Hu X or 5). n + n’ indicate the extent of the consensus sequence around the *nic* site; mismatch position with respect to the consensus sequence is indicated in brackets.

**(3)** Coordinates 174 to 190 according to [8]
